# Depression, Anxiety, and Stress Scale-21 (DASS-21): Further psychometric exploration using robust item response theory and classical theory measures among university students

**DOI:** 10.1371/journal.pone.0325238

**Published:** 2025-07-28

**Authors:** Md Dilshad Manzar, Mohammed Salahuddin, Dejene Nureye, Faizan Z. Kashoo, Majumi M. Noohu, Jazi Shaydied Alotaibi, Majed Sulaiman Alamri, Mark D. Griffiths

**Affiliations:** 1 Department of Primary Nursing Care, College of Nursing, Majmaah University, Al Majmaah, Saudi Arabia; 2 Department of Pharmaceutical Sciences, School of Pharmacy, Notre Dame of Maryland University, Maryland, United States of America; 3 School of Pharmacy, College of Medicine and Health Sciences, Mizan-Tepi University, Mizan-Aman, South West Ethiopia Peoples’ Region, Ethiopia; 4 Research Unit of Neuro-inflammatory and Cardiovascular Pharmacology, Faculty of Science, University of Dschang, Dschang, Cameroon; 5 Department of Physical Therapy and Health Rehabilitation, College of Applied Medical Sciences, Majmaah University, Al Majmaah, Saudi Arabia; 6 Centre for Physiotherapy and Rehabilitation Sciences, Jamia Millia Islamia, New Delhi, India; 7 Department of Nursing, College of Applied Medical Sciences, Majmaah University, Al Majmaah, Saudi Arabia; 8 Department of Medical, Surgical and Critical Care Nursing, College of Nursing, University of Hafr Albatin, Hafr Albatin, Saudi Arabia; 9 International Gaming Research Unit, Psychology Department, Nottingham Trent University, Nottingham, United Kingdom; University of Birjand, IRAN, ISLAMIC REPUBLIC OF

## Abstract

**Background:**

Psychometric validity of the 21-item Depression Anxiety and Stress Scale (DASS-21) has not been studied among Ethiopians. This study investigated the psychometric properties of the DASS-21 using Item Response Theory (IRT) and classical theory among Ethiopian university students.

**Methods:**

A cross-sectional study was conducted with 364 Ethiopian university students. Participants completed a socio-demographic questionnaire, the Hamilton Anxiety Scale (HAM-A), and the DASS-21.

**Results:**

Factor analysis and measures of quality of factor estimates, construct stability, and robustness of unidimensionality supported a one-factor structure. Item parameters were satisfactory for all but three items using both CTT and IRT. Some CTT parameters indicated deviation from unidimensionality for three items. Two items had poor communality, and IRT infit/outfit values above 1.4. The findings indicated adequate divergent validity (weak-moderate correlation) for the general distress measure of DASS-21 with respect to HAM-A, excellent reliability (McDonald’s ω and Cronbach’s α both 0.93), and item-level measurement invariance across sexes.

**Conclusion:**

The psychometric validity of the DASS-21 among Ethiopian university students was robust, as shown by analysis using classical and item response theory measures. However, some item-level psychometric characteristics for two items were sub-optimal in both CTT and IRT.

## Introduction

Depression, anxiety, and perceived stress are common mental health problems among university students worldwide [1,2,3]. A national study in the US on mental health showed more than 60% of university students suffered from at least one mental health problem [4]. Mental health issues predict lower academic success, with a diagnosis of depression related to a twofold increase in the probability of dropping out of midwestern public universities in the US [5].

Students might experience significant stressors in the academic setting, which is often due to rigorous coursework, exam stress, and worries concerning future job prospects [6,7]. Moreover, the difficulties of adjusting to a new social environment, moving away from family, and dealing with financial restraints can all contribute to feelings of anxiety and depression [8]. Numerous studies report over 75% of university students experience moderate to severe psychological distress [9]. Therefore, it is important to identify effective screening tools for those who are at risk and need timely support. The 21-item Depression, Anxiety, and Stress Scale (DASS-21) has shown potential in assessing these emotional states, and its validation using rigorous psychometric approaches may further help establish applicability among university students [10,11].

The DASS-21 is a widely used psychometric instrument, comprising three seven-item subscales that assess the severity of symptoms related to depression, anxiety, and stress. The depression subscale assesses dysphoria, despair, anhedonia, lethargy, and apathy [10,11]. The anxiety subscale assesses autonomic arousal, situational anxiety, and subjective perception of anxious feelings [10,11]. The perceived stress subscale assesses chronic nonspecific arousal levels, including difficulties in achieving a state of relaxation, heightened nerve arousal, increased irritability, excessive reactivity, and a lack of tolerance. Total scores are derived by summing the scores of each of the three subscales [10,11].

The DASS-21 is widely used by mental health professionals to identify and assess the severity of these three mood states among individuals. It has good psychometric properties with satisfactory validity and reliability parameters [10,11]. However, most of the studies investigating psychometric characteristics of the DASS-21 have had one or more of these limitations: (i) presuming the presence of continuous data distribution [12,13], (ii) performing factor structure analysis with no/less diligence to data suitability parameters for factor analysis [12,13], and (iii) limited use of item response theory [14]. Moreover, the psychometric characteristics of DASS-21 have not been extensively studied in Africa. Therefore, the present study examined the psychometric properties of the DASS-21 using robust statistical methods employing methods suitable for Likert-scale scores, tests verifying assumptions of factor analysis, and appropriate item response theory-based parameters (i.e., a polytomous rating scale model) among a sample of Ethiopian university students.

## Methods

### Participants, procedure, study design, and ethics

The present cross-sectional study employed a convenience sampling method. Researchers who were faculty members of Mizan-Tepi University’s College of Medicine and Health Sciences administered the survey during Oct-Dec, 2018. The study involved distributing information about the study through university channels, such as emails, flyers, notice boards and classroom announcements, providing detailed information about objectives, procedures, and confidentiality measures. Interested participants were asked to contact the research team for further information or participation. They were then provided with information regarding informed written consent, and given the chance to ask questions about the study. The contact information of an investigator was shared with the participants to address any inquiries or seek more information. Participants were informed that their participation was entirely voluntary and that they had the right to withdraw at any time without any negative consequences. Participants did not receive any rewards for taking part in the study and those participants who provided informed written consent were then included in the study.

The source population comprised a cohort of 500 students from the Mizan health campus of MTU in Ethiopia. The inclusion criteria comprised (i) being students who were registered in courses at MTU at the time of data collection, and (ii) being at least 18 years of age. The final sample population comprised 364 university students who agreed to participate in the present study.

The research study received approval from the Ethical Committee of the College of Medicine and Health Sciences at MTU in Ethiopia. The participants who took part in the study completed the DASS-21, the Hamilton Anxiety Scale, and a socio-demographic information questionnaire in the English language (see next section for details). The students who participated in academic activities at MTU and other federal universities in Ethiopia possess sufficient English language skills because English is the primary medium of instruction at these institutions.

### Instruments

#### Depression, anxiety, and stress scale-21 (DASS-21).

The 21-item DASS-21 is a shorter version of the 42-item DASS-42 [10]. Each item in the scale is evaluated using a numerical Likert scale ranging from 0 to 3, where 0 indicates that the item did not apply to the participant at all, and 3 indicates that the item applied to the participant very much or most of the time. The DASS-21 comprises three distinct subscales, namely depression, anxiety, and stress, each consisting of seven items. The scores of each item are combined to calculate subscale scores, which can range from 0 to 21. Higher scores on the DASS-21 subscales are indicative of a progressive escalation in the intensity of symptoms related to depression, anxiety, and stress. The psychometric properties of the DASS-21 in the present study are reported in the Results section.

#### Hamilton Anxiety Scale (HAM-A).

The 14-item Hamilton Anxiety Rating Scale (HAM-A) was used to assess the severity of anxiety symptoms [15]. The rating scale for all items ranges from 0 (indicating the absence of anxiety) to 5 (indicating a high level of anxiety). The individual scores of the items are summed together to provide a cumulative score, which ranges from 0 to 56. Higher scores are indicative of a greater severity of anxiety symptoms. The internal consistency (Cronbach’s alpha) ranged from 0.77–0.92 in previous studies [15].

#### Socio-demographic questions.

Information was collected regarding age, gender, and number of years of university education.

### Data analysis

Four software packages were used to analyze the dataset in the present study, (i.e., SPSS 23.0, JASP 0.17.0.0, JAMOVI 2.3.18, and Factor 12.03.02). Socio-demographic characteristics and DASS-21 item properties were analyzed using descriptive methods. Internal reliability and classical item theory parameters such as Cronbach’s alpha, McDonald’s omega, item-rest correlations, skewness, and kurtosis were calculated using JAMOVI 2.3.18.

Several statistical measures were used to assess that the DASS-21 item scores in the study sample satisfied the assumptions for factor analysis including the Kaiser-Meyer-Olkin (KMO), polychoric correlation coefficient matrix of DASS-21 items scores, Bartlett’s test of sphericity, determinant of the correlation matrix, and Mardia’s test of skewness and kurtosis [16,17]. Finally, as the sample size in this study exceeded 200 with multiple indicators, i.e., more than three indicators for every latent variable (based on the maximum number of factors for the DASS-21 scale recorded in the literature, i.e., three), therefore, the sample size is adequate to satisfy the condition of zero convergence failures [18].

Semi-confirmatory factor analysis (SCFA) was performed by Factor 12.03.02 on polychoric correlation matrix with the following settings: robust diagonally weighted least squares (RDWLS) estimation, robust Promin rotation [19], factor estimates based on linear model with bootstrap sampling (n = 500), and hot-deck multiple imputation method [20]. Factor extraction was based on (i) eigenvalue of more than 1, (ii) cumulative variance of more than 40%, (iii) scree plot, (iv) parallel analysis, and (v) Hull method [16,17,21],.

The performance of the factor structure model of the DASS-21 was assessed using multiple fit indices such as the goodness of fit index (GFI), non-normed fit index (NNFI), comparative fit index (CFI), root mean square error of approximation (RMSEA), weighted root mean square residual (WRMR), and robust mean and variance-adjusted chi-square [16,17]. For an excellent fit of the model, it was required to have a value of 0.95 and above for GFI, CFI, and NNFI. Similarly, the model was required to have a value of 0.05 or less for WRMR and RMSEA [16,22]. Measures to assess the quality of factor estimates such as the factor determinacy index (FDI), expected posteriori marginal reliability, sensitivity ratio (SR), and the expected percentage of true differences (EPTD) were determined. For an adequately reliable quality of factor estimates, it is advised to employ factor scores with FDI values above.90, marginal reliabilities above.80, SR above 2, and EPTDs over 90% [23]. Measures to establish unidimensionality of factor structure such as UniCo (unidimensional congruence) and I-Unico (item unidimensional congruence), ECV (explained common variance) and I-ECV (item explained common Variance), MIREAL (mean of item residual absolute loadings) and I-REAL (item residual absolute loadings) were also determined. For a reliable one-factor solution, it is advised that UniCo and I-Unico values are above.95, ECV and I-ECV are above.85, and MIREAL and I-REAL are lower than 0.30 [23].

Measures to establish construct replicability such as H-latent, and H-observed values were determined. High H-values (H-latent, and H-observed) (>.80) point to a latent variable that is well-defined and more likely to remain constant across studies, whereas low H-values point to a latent variable that is poorly defined and more likely to vary over time [24].

As aforementioned, all 21 items of the DASS-21 are scored on a 4-point Likert scale with a range of 0–3 with identical response categories. Therefore, the polytomous rating scale model was used as it is based on common threshold parameters for all items and invariant response format [25]. eRm R package in the snowIRT program of JAMOVI 2.3.18 was used to determine marginal maximum likelihood estimates of item difficulty, an information-weighted fit statistic (infit) mean square (MnSq) and outlier-sensitive fit statistic (outfit) MnSq, and thresholds (τi1, τi2, τi3, and τi4). Graphical measures of item parameters such as the Wright map, person-item distribution, and Item characteristic curves (ICCs) were performed [25]. The difNLR package of R in snowIRT program of JAMOVI 2.3.18 was used to perform differential item function (DIF) test, and generalized logistic regression models for DIF estimation [26].

## Results

### Participants’ characteristics

Most of the participating students recorded their age to be 18–25 years (Table 1) and over two-thirds of the study participants identified themselves as male (67%). Over three-fifths of the study participants were enrolled in the second year of their university education (62.1%). Average scores on the DASS-21 subscales were 13.0 out of 21 (SD ± 9.02) for depression, 13.19 out of 21 (SD ± 9.06) for anxiety, and 13.30 out of 21 (SD ± 8.65) for stress.

**Table 1:** Participants characteristics of the university students.

Characteristics	Mean ± SD/ Frequency
Age	
18–25 years	335 (92.0%)
26 years and above	26 (7.1%)
Did not report	3 (0.8%)
Gender	
Male	244(67.0%)
Female	120(33.0%)
Duration of university education (In years)	
1	73 (20.1%)
2	226 (62.1%)
3	25 (6.9%)
4	39 (10.7%)
5	1 (0.3%)
DASS-21	
Depression subscale	13.0 ± 9.02
Anxiety subscale	13.19 ± 9.06
Stress subscale	13.30 ± 8.65

SD: Standard deviation; DASS-21: Depression, Anxiety and Stress Scale – 21 Items

### Factor analysis

#### Suitability and adequacy of the DASS-21 data for factor analysis.

All the inter-item polychoric correlation coefficients were significant, and most were above 0.3 (174 out of 200) (Table S1 in S1 File). The KMO criterion overall value was 0.939 and KMO for individual items of the DASS-21 ranged from 0.889 to 0.964 (Table 2 and Table S2 in S1 File). The correlation matrix of DASS-21 items scores differed significantly from an identity matrix (Bartlett’s test of sphericity: χ^2^(210) = 3314.75, *p* < 0.001). The determinant of the correlation matrix was more than 0.00001 (Table S2 in S1 File). Assumptions of the multivariate normality were violated: Mardia’s kurtosis (χ^2 ^= 29.923, *p* < .001) (Table S2 in S1 File). Two items had communality scores below 0.2 (Item 2, and Item 3) (Table 2).

**Table 2:** Closeness to dimensionality measures, communality and correlation coefficients with HAM-A of the DASS-21 scores in university students.

Items of the DASS-21	I-UniCo	I-ECV	IREAL	Communality (h^2^)^¥^	correlation coefficients with HAM-A ^#, *^	Normed MSA	if Item Deleted		Correlation coefficients
Cronbach’s α	McDonald’s ω	Item-rest^#, *^
DASS_1	0.960	**0.774**	**0.353**	0.405	0.374	0.928	0.90	0.90	0.51
DASS_2	**0.414**	**0.313**	**0.650**	**0.176**	0.237	0.874	0.91	0.91	0.36
DASS_3	**0.591**	**0.423**	**0.527**	**0.180**	0.162	0.919	0.91	0.91	0.38
DASS_4	0.999	0.967	0.124	0.416	0.324	0.940	0.90	0.90	0.53
DASS_5	1.000	0.974	0.107	0.400	0.365	0.950	0.90	0.90	0.55
DASS_6	0.994	0.902	0.189	0.295	0.285	0.940	0.91	0.91	0.47
DASS_7	1.000	0.970	0.115	0.408	0.364	0.936	0.90	0.90	0.54
DASS_8	0.994	0.904	0.206	0.366	0.347	0.956	0.90	0.90	0.53
DASS_9	0.999	0.962	0.137	0.456	0.429	0.940	0.90	0.90	0.59
DASS_10	1.000	0.993	0.058	0.436	0.435	0.948	0.90	0.90	0.56
DASS_11	1.000	0.998	0.031	0.470	0.430	0.926	0.90	0.90	0.60
DASS_12	0.993	0.896	0.214	0.372	0.414	0.924	0.90	0.90	0.52
DASS_13	1.000	0.983	0.093	0.474	0.546	0.965	0.90	0.90	0.61
DASS_14	0.996	0.916	0.209	0.460	0.486	0.904	0.90	0.90	0.59
DASS_15	1.000	0.996	0.046	0.502	0.406	0.960	0.90	0.90	0.61
DASS_16	1.000	0.991	0.064	0.399	0.352	0.945	0.90	0.90	0.55
DASS_17	0.998	0.944	0.159	0.401	0.328	0.937	0.90	0.90	0.52
DASS_18	0.996	0.914	0.209	0.446	0.398	0.947	0.90	0.90	0.56
DASS_19	0.999	0.954	0.131	0.330	0.372	0.944	0.91	0.90	0.50
DASS_20	0.997	0.933	0.192	0.505	0.374	0.943	0.90	0.90	0.59
DASS_21	0.995	0.910	0.222	0.487	0.388	0.941	0.90	0.90	0.60

* p < 0.01, ^#^ Spearman’s correlation coefficient, I-UniCo: Item Unidimensional Congruence; I-ECV: Item Explained Common Variance, I-REAL: Item Residual Absolute Loadings

#### Factor analysis: Semi-confirmatory factor analysis of the DASS-21.

The results of the factor extraction measure in the exploratory factor analysis yielded disparate results. Kaiser’s criteria of the eigenvalue of more than 1 found two factors. Cumulative variance above the 40% criterion, scree test, and parallel analysis (both based on principal component analysis (PCA), and the minimum rank factor analysis (MRFA)) indicated a one-factor solution (Fig S1 in S1 File, and Table 3). The one-factor solution was also supported by Hull method, where it showed highest scree test values. The scree test values were highest for a one-factor solution when calculated using three different fit indices; robust RMSEA, robust CFI, and common part accounted for (CAF).

**Table 3:** Summary of the factor extraction measures used in exploratory factor analysis of the Depression Anxiety Stress (DASS-21) scale scores in university students.

Number of Factors	Eigenvalue	Cumulative VA^*^	Above point of inflection on Scree plot	PA based on minimum rank			Decision to extract			
				Real-data VE^*^	Mean of random VE^*^	95^th^ percentile of random VE^*^	Kaiser’s criteria (Eigenvalue≥1)	Cumulative VE rule (>40%)	Scree test	Real data VE> random VE
1	8.866	42.22	Yes	49.501^*^	9.954	11.016	√	√	√	√
2	1.242	48.14	No	6.400	9.150	9.914	√	Χ	Χ	Χ
3	0.978	52.80	No	5.256	8.555	9.271	Χ	Χ	Χ	Χ

√ indicates extraction criteria fulfilled, Χ indicates otherwise

* Values in percentage; VE: Variance Explained; PA: Parallel analysis

The model fit indices showed that a one-factor structure had adequate fit: GFI (1.00), NNFI (0.995), CFI (0.995) were above 0.99; WRMR (0.049), and RMSEA (0.028) were below 0.05; and the chi-square test was significant, χ^2^(189) =239.344, *p* = .008). The factor loading had a range of 0.419 to 0.710 with an average of 0.627 for the DASS-21 item scores among the study participants (Table S3a-d in S1 File). Both 2-factor and 3-factor solutions had better fit indices (Table 4), but both had poor factor loading across second and third factors (Table S3a-d in S1 File). Similarly, a bifactor model with three first-level factors also showed very low factor loadings for all first-level factors.

**Table 4:** Fit indices of the Depression, Anxiety and Stress Scale – 21 Items (DASS-21) in the Ethiopian university students.

Model	GFI	NNFI	CFI	WRMR	RMSEA (90% CI)^*^	χ^2^	df	p-value	χ^2^/df
1-Factor	1.00	.995	.995	.049	.028 (.013 −.036)	239.344	189	.008	1.266
2-Factor	1.00	0.998	0.999	0.0406	.015 (.00 −.027)	181.454	169	.242	1.074
3-Factor	1.00	1.000	0.999	0.0355	.00 (.00 −.023)	147.464	150	.543	0.983

* Not calculated by the software used for factor analysis, Factor 10.08.06 but calculated manually. CI: Confidence interval; GFI: Goodness of Fit Index; NNFI: Non-normed Fit Index; CFI: Comparative Fit Index; WRMR: Weighted Root Mean Square Residual, RMSEA: root mean square error of approximation

All fit indices were estimated for Robust Diagonally Weighted Least Squares extraction method (RWDLS) with Robust Promin

#### Robustness of factor score estimates, and unidimensionality of the DASS-21.

The values of measures of quality of factor estimates for FDI, EPMR, SR, and EPTD were 0.967, 0.936, 3.825, and 94.4% respectively (Table S2 in S1 File). The values of measures of construct stability for H-latent and H-observed were 0.936 and 0.917, respectively (Table S2 in S1 File). Because the DASS-21 was found to be have a one-factor structure, measures to assess robustness of unidimensionality were assessed. The values of the overall measures for UniCo, ECV, and MIREAL were 0.949, 0.877, and 0.192, respectively (Table 2).

### Classical theory-based item analysis parameters of the DASS-21

There was no major pattern in the missing values for the DASS-21 items scores among study participants. All items had missing values (range: 1.4% to 2.2%), and 6.9% students did not answer at least one of the items with a low overall percentage of missing values (1.6%) (Table 5). The absolute values of skewness statistics had a range of 0.42 to 0.98, and absolute values of kurtosis statistics had a range of 0.02 to 0.96 (Table 5).

**Table 5:** Distribution properties: Skewness, kurtosis, percentage distribution-ceiling/floor effect parameters of the Depression Anxiety Stress (DASS-21) scale scores in university students.

Items of the DASS	Mean	SD	Skewness			Kurtosis			Percentage distribution across item scores				
			Statistic	SE	z	Statistic	SE	z	0	1	2	3	Missing values
DASS_1	0.80	0.92	0.98	0.13	7.56	0.02	0.26	0.07	46.7	31.9	12.4	7.1	1.9
DASS_2	0.84	0.94	0.93	0.13	7.17	−0.09	0.26	−0.37	44.8	33.2	12.6	8.0	1.4
DASS_3	0.96	1.05	0.63	0.13	4.88	−0.95	0.26	−3.69	45.6	21.2	21.2	10.2	1.9
DASS_4	0.80	0.97	0.88	0.13	6.85	−0.43	0.26	−1.67	51.1	23.4	17.3	6.9	1.4
DASS_5	0.98	0.94	0.60	0.13	4.66	−0.62	0.26	−2.42	37.1	34.1	19.8	7.4	1.6
DASS_6	1.08	0.95	0.45	0.13	3.49	−0.80	0.26	−3.11	32.4	34.3	23.1	8.5	1.6
DASS_7	0.89	0.94	0.77	0.13	6.00	−0.39	0.26	−1.52	42.0	32.7	16.2	7.4	1.6
DASS_8	0.99	0.94	0.55	0.13	4.24	−0.71	0.26	−2.77	36.5	33.2	21.4	7.1	1.6
DASS_9	1.10	1.00	0.42	0.13	3.29	−0.96	0.26	−3.73	34.6	29.9	24.2	9.9	1.4
DASS_10	0.93	0.96	0.70	0.13	5.46	−0.56	0.26	−2.16	40.7	31.9	17.6	8.2	1.6
DASS_11	0.97	0.99	0.67	0.13	5.16	−0.68	0.26	−2.63	40.1	30.5	18.1	9.3	1.9
DASS_12	0.95	0.97	0.69	0.13	5.32	−0.58	0.26	−2.27	40.1	32.1	17.9	8.5	1.4
DASS_13	0.86	0.94	0.79	0.13	6.14	−0.42	0.26	−1.63	44.2	29.9	16.8	6.9	2.2
DASS_14	1.06	1.00	0.52	0.13	4.01	−0.86	0.26	−3.35	36.0	30.8	21.2	10.2	1.9
DASS_15	0.91	0.96	0.74	0.13	5.75	−0.50	0.26	−1.95	42.0	31.3	17.0	8.0	1.6
DASS_16	0.88	0.98	0.82	0.13	6.35	−0.46	0.26	−1.80	45.3	28.6	15.7	8.8	1.6
DASS_17	0.94	0.94	0.60	0.13	4.63	−0.72	0.26	−2.81	40.4	30.5	21.4	6.3	1.4
DASS_18	0.88	0.89	0.73	0.13	5.65	−0.32	0.26	−1.24	39.8	35.7	16.8	5.5	2.2
DASS_19	1.06	0.99	0.45	0.13	3.49	−0.95	0.26	−3.68	36.3	29.1	24.2	9.1	1.4
DASS_20	0.97	0.99	0.60	0.13	4.62	−0.84	0.26	−3.25	41.5	27.2	21.4	8.5	1.4
DASS_21	0.96	1.10	0.73	0.13	5.67	−0.89	0.26	−3.47	47.5	21.7	15.1	14.3	1.4

* *p *< 0.05; ^*^ *p *< 0.001; SD: Standard deviation; SE: Standard Error

Item scores showed a floor effect but no ceiling/floor effect was seen in total scores. With regards the subscale scores: (i) on the depression subscale, 9.6% recorded the lowest score, and none recorded the highest score, (ii) on the anxiety subscale, 8.2% recorded the lowest score, and 0.3% recorded the highest score, and (iii) on the stress subscale, 7.7% recorded the lowest score, 0.3% recorded the highest score. For the DASS-21 total score, 4.7% recorded the lowest score, and none recorded the highest score (Table 5) [27,28]. The values of individual item-Unico (I-Unico) were above 0.95 except for two items (Item 2 and Item 3) (Table 2). The values of individual item-ECV (I-ECV) were above 0.85 except for three items (Items 1–3; Table 2). The values of individual item-REAL (IREAL) were below 0.3 except for three items (Items 1–3; Table 2).

### Internal consistency and item discrimination

The internal consistency measures of McDonald’s omega and Cronbach’s α for the overall DASS-21 were both 0.93. There was little variation in McDonald’s omega and Cronbach’s α if items were deleted one at a time (Table 2). The item-rest correlation values ranged from 0.36 to 0.61 (Table 2).

### Divergent validity: correlation coefficient of DASS-21 with the measure of HAM-A

All the correlation coefficients between DASS-21 item scores and the HAM-A total score were significant and ranged from 0.16 to 0.55.

### DASS-21 item analysis: rasch rating scale model parameters

**Fig 1:**
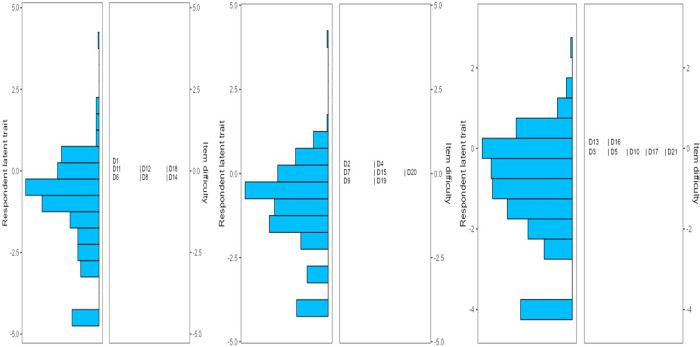
Wright map Person-item distribution for individual items of the Depression Anxiety Stress Scale (DASS-21). D1 to D21 are items of the DASS-21.

The rating scale parameters of DASS-21 such as difficulty level (range: 0.656–1.129), infit (range: 0.846–1.466), outfit statistics (range: 0.807–1.589), and threshold estimates are shown in Table 6. Three threshold estimates (τi1, τi2, and τi3) for all the 21 items of the DASS-21 were ordered. Scores for all the 21 items of the DASS-21 had an expected pattern across response levels. For example, for latent dimension 2.0 (Fig S2 in S1 File), all items had a similar probability of recording second response level (~38%). The range and spread of the person ability (i.e., general distress) was wide, while item difficulty level had a narrow range (Fig 1). The DIF test was non-significant for all 21 items of the DASS-21 (Table 7).

**Table 6:** Summary of item difficulty, Polytomous Mean-Square Fit Statistics (infit, outfit), and threshold(τi) statistics of the Rating Scale Model: Depression Anxiety Stress (DASS-21) scale scores in university students.

Item	Severity	(SE)	Outfit MnSq	Infit MnSq	Threshold 1 (τi1)	Threshold 2 (τi2)	Threshold 3 (τi3)
DASS_1	1.155	0.072	1.020	0.944	0.241	1.018	1.680
DASS_2	1.098	0.071	1.295	1.589	0.185	0.962	1.620
DASS_3	0.898	0.069	1.466	1.534	−0.011	0.766	1.430
DASS_4	1.129	0.072	1.094	1.061	0.216	0.992	1.650
DASS_5	0.846	0.068	0.941	0.920	−0.062	0.715	1.380
DASS_6	0.669	0.067	1.026	1.042	−0.236	0.541	1.200
DASS_7	0.999	0.070	0.953	0.913	0.088	0.865	1.530
DASS_8	0.814	0.068	0.944	0.922	−0.094	0.683	1.350
DASS_9	0.656	0.067	0.914	0.891	−0.249	0.528	1.190
DASS_10	0.912	0.069	0.989	0.971	0.003	0.779	1.440
DASS_11	0.898	0.069	0.911	0.869	−0.011	0.766	1.430
DASS_12	0.912	0.069	1.012	1.036	0.003	0.779	1.440
DASS_13	1.038	0.070	0.909	0.853	0.126	0.903	1.570
DASS_14	0.723	0.067	0.952	0.911	−0.183	0.594	1.260
DASS_15	0.950	0.069	0.889	0.819	0.040	0.817	1.480
DASS_16	1.033	0.070	1.066	0.999	0.121	0.898	1.560
DASS_17	0.903	0.069	0.964	0.988	−0.007	0.770	1.430
DASS_18	1.008	0.070	0.846	0.807	0.097	0.874	1.540
DASS_19	0.728	0.067	1.088	1.046	−0.179	0.598	1.260
DASS_20	0.842	0.068	0.921	0.882	−0.067	0.710	1.370
DASS_21	0.888	0.069	1.212	1.099	−0.021	0.756	1.420

Based on eRm R package

**Table 7:** Differential item function (DIF) test: Depression Anxiety Stress (DASS-21) scale scores in university students across gender groups.

Items of the DASS-21	Uniform DIF estimate			Non-uniform DIF estimate		
	Likelihood ratio Chi-square statistics	Unadjusted p-value	Adjusted p-value	Likelihood ratio Chi-square statistics	Unadjusted p-value	Adjusted p-value
DASS_1	1.4788	0.224	0.764	0.20059	0.654	0.973
DASS_2	0.0471	0.828	0.87	4.84104	0.028	0.204
DASS_3	0.5779	0.447	0.838	0.14999	0.699	0.973
DASS_4	0.184	0.668	0.838	0.08944	0.765	0.973
DASS_5	1.2663	0.26	0.764	3.84578	0.05	0.262
DASS_6	0.1077	0.743	0.838	7.8283	0.005	0.108
DASS_7	1.6082	0.205	0.764	0.31418	0.575	0.973
DASS_8	0.6291	0.428	0.838	0.00117	0.973	0.973
DASS_9	3.9024	0.048	0.764	0.48433	0.486	0.973
DASS_10	1.5255	0.217	0.764	0.01094	0.917	0.973
DASS_11	0.0946	0.758	0.838	0.00821	0.928	0.973
DASS_12	0.3215	0.571	0.838	4.76271	0.029	0.204
DASS_13	0.1309	0.718	0.838	0.08434	0.772	0.973
DASS_14	0.8409	0.359	0.838	0.02273	0.88	0.973
DASS_15	2.6452	0.104	0.764	0.11352	0.736	0.973
DASS_16	0.1101	0.74	0.838	0.00796	0.929	0.973
DASS_17	0.0173	0.895	0.895	0.08512	0.77	0.973
DASS_18	1.1146	0.291	0.764	0.12928	0.719	0.973
DASS_19	0.1606	0.689	0.838	2.42382	0.12	0.502
DASS_20	0.2195	0.639	0.838	0.64268	0.423	0.973
DASS_21	1.5407	0.215	0.764	0.07482	0.784	0.973

## Discussion

The present study showed that the DASS-21 had adequate validity measures supported by a robust psychometric investigation among Ethiopian university students. In summary, the DASS-21 was found to have a one-factor structure with robust factor score estimates, and construct stability measures, adequate item parameters based on both CTT and IRT, divergent validity, and high internal consistency in the study population. The present study is one of the few that has examined the DASS-21 employing all of these (i.e., psychometric methods suitable for Likert-scale scores), tests verifying assumptions of factor analysis, and appropriate item response theory-based parameters (i.e., the polytomous rating scale model).

### Factor analysis

#### Suitability and adequacy of the DASS-21 data for factor analysis.

As items of the DASS-21 were scored on a Likert scale, and structural validity was assessed using a polychoric correlation matrix [29]. Moreover, the implementation of polychoric correlation was necessitated by the violation of multivariate normality criteria [30]. However, many of the previous studies reporting the factorial validity of the DASS-21 have used a correlation matrix for factor analysis [12,13]. Such an approach has statistically less rigor, more so, because some of these studies did not report the outcome of tests of multivariate normality [12]. Following the commonly used recommendation of employing multiple parameters to indicate the suitability of data to perform factor analysis, Kaiser-Meyer-Olkin (KMO), polychoric correlation coefficient matrix, Bartlett’s test of sphericity, communality, and determinant of the correlation matrix were used [16,17]. However, some of the previous studies reporting the structural validity of the DASS-21 did not report the data suitability and adequacy measures [12,13,31]. Indeed, recent systematic reviews and meta-analyses have highlighted that there is a common problem of non-reporting, and/or under-reporting of data assumptions testing in studies investigating structural validity ( [16,17].

Therefore, in the present study, this important caveat was adequately addressed by the use of reporting univariate/multivariate normality, methods to manage violation of multivariate normality, missing value management, reporting of KMO, Bartlett’s tests, communality, use and reporting of the polychoric correlation matrix, and determinant score. To the best of the authors’ knowledge, the present study is the first to examine the structural validity of DASS-21 that has reported all these data screening and assumption testing procedures. Two items with sub-threshold values of communality were retained in the model because these performed adequately on other parameters such as robust measures of IRT, and other CTT measures such as Cronbach’s alpha/McDonald’s omega if item deleted, factor loading, and item-rest correlation [32].

#### Semi-confirmatory factor analysis of the DASS-21.

In the present study, SCFA, a novel variant approach which is partially explorative, and partially confirmatory was used. This approach is easily implemented in freely accessible software (i.e., Factor by Universitat Rovira i Virgili) [34]. In this approach, a suitable factor structure was explored along with an estimation of its fit indices. In addition, measures assessing the robustness of factor score estimates and construct stability were estimated. In the present study, a one-factor solution of the DASS-21 was viewed as suitable because all except one factor measure (Kaiser’s criteria of eigenvalue of more than 1) including robust parallel analysis (estimated using two methods: PCA and MRFA) and the Hull method (scree test values calculated using three different fit indices: robust RMSEA, robust CFI, and CAF), as well as all except one fit index (chi-square test) were adequate. Alternative factor structures such as 2-factor, 3-factor [35], and bifactor models with three first-level factors [36] were deemed not suitable for the study sample because of very low factor loading values.

Moreover, in the present study, multiple measures of factor extraction, and fit indices were employed [16,17], and is an important strength. All the individual factor loadings were adequate and explained a good level of variance overlap (except two items; items 2 and 3) [37].

Similar to the present study, the unidimensional construct of the DASS-21 has been found among Egyptian drug users [35], Latin-speaking American college students [38], school-going Australian children and adolescents [39], and college students in eight countries spread over four continents [40]. It is noteworthy to observe that three of these previous studies involved study populations with similar or nearly similar age groups [35,39,40]. Therefore, together with the findings of present study, unidimensionality of the DASS-21 has been found among young adults across six continents. Consequently, the unidimensional construct of the DASS-21 may be used to screen a general distress factor among young adults.

#### Robustness of factor score estimates, and unidimensionality of the DASS-21.

To the best of the authors’ knowledge, the present study is the first to assess measures of quality and effectiveness of factor estimates, construct replicability, and stability of unidimensionality for the DASS-21. Therefore, because these results are novel, no comparison with previous studies can be reported. All the four measures of quality and effectiveness of factor estimates (i.e., FDI, EPMR, SR, and EPTD) were in the ideal range suggesting that DASS-21 factor scores are useful for individual assessment [23]. Further support for the construct validity was indicated by adequate value of construct replicability parameters, H-latent, and H-observed [24].

### Classical theory-based item analysis parameters of the DASS-21

The findings of the present study showed that there was no statistically significant deviation from the univariate distribution for the items, factors, and total scores of the DASS-21. This suggests that the distribution of scores conformed to a pattern seen in a general population [41]. This aspect enhances the overall validity of the study’s findings. Moreover, the lack of a ceiling or floor effect observed in the DASS-21 total score indicates that it can distinguish between groups [28]. The presence of floor effects in all DASS-21 item scores could potentially be attributed to the non-clinical composition of the research population, specifically consisting of emerging adults attending Ethiopian universities [42].

There was some concern about deviation from unidimensionality for three item scores (Items 1–3). Moreover, it is noteworthy that two of these items also had poor communality and low values for I-UniCo, I-ECV and IREAL. However, these items were not deleted because they performed adequately on other parameters, such as the robust measures of IRT and other CTT measures, such as Cronbach’s alpha/McDonald’s omega if item deleted, and item-rest correlation [32,33]. All but two items had factor loading less than 0.54; this means that the overlapping variance was 30%, which is considered good [37]. Two items (items 2 and 3) had factor loading values that indicated a poor-to-fair level of overlapping variance [37]. As statistical evidence was mixed for these items, therefore, it would be interesting to explore modifications such as the rephrasing of these items to better reflect unidimensional congruence [23]. In summary, all three of these broad categories of measures further supported the validity of the one-factor model of the DASS-21, except for some concerns for two items in this study population.

### Internal consistency and item discrimination

In the present study, the DASS-21 had an excellent level of internal consistency as assessed by McDonald’s omega and Cronbach’s α, both of which yielded a coefficient of 0.93 for both measures [43]. McDonald’s omega and Cronbach’s α were calculated because there was no major concern of deviation from univariate normality for all the individual item scores of the DASS-21 [44,45]. Camacho et al. 2016 reported a McDonald’s omega of 0.95 among Latin-speaking American university students [38]. Similarly, Patrick et al. 2010 reported Cronbach’s alpha values of 0.83 to 0.96 among school-going Australian children and adolescents [39]. Therefore, the reliability of the DASS-21 scale is excellent among university students and children. All the items contributed almost equally to the reliability as evidenced by non-significant changes in McDonald’s omega and Cronbach’s α if items were deleted one at a time.

### Divergent validity: correlation of DASS-21 scores with the measure of HAM-A

In the present study, the divergent validity of the DASS-21 (assessed with respect to the expert-administered HAM-A) was adequate because all correlation coefficients were weak to moderate, and none were strong. This is expected because DASS-21 was found to have a 1-factor structure possibly measuring a global distress score rather than three separate properties of depression, anxiety, and stress. Therefore, its correlation with a specific measure of anxiety, i.e., HAM-A, was weak to moderate. The divergent validity outcome further supports the 1-Factor structure of the DASS-21. Similar to previous studies, the DASS-21 was found to have a 1-Factor structure measuring a general distress rather than three distinct measures of depression, anxiety, and stress in this study population [35,38–40]. Therefore, the weak-moderate correlation between the DASS-21—a measure of general distress, not anxiety—and HAM-A scores (a measure of anxiety severity in clinical settings) shows the degree of relatedness between two distinct but related constructs. Therefore, this result gives adequate support to the divergent validity of DASS-21 in this study population.

### DASS-21 item analysis: rasch rating scale model parameters

To the best of the authors’ knowledge, the present study is the first to employ such an extensive and elaborate set of IRT measures including difficulty level, MNSQ infit/outfit, threshold estimates, DIF across sex, ICCs, and Wright map for assessing validity of DASS-21. Six items (i.e., Items 1, 2, 4, 13, 16, and 18) had a difficulty level above 1. This possibly implied that symptoms assessed with these items were likely to be endorsed with higher category responses only by those students who had high level of general distress as assessed by the DASS-21 [46]. When an item’s MNSQ infit/outfit value exceeds 1.4, it suggests that the items do not belong to the same construct as other items in the domain/tool. MNSQ values around 1.0 are optimal, with values less than 0.6 potentially suggesting item redundancy [47]. Two items (Items 2 and 3) had MNSQ infit/outfit values above 1.4, implying a non-fit with the unidimensional construct of the DASS-21 [47]. Not surprisingly, these two items, also had concerns regarding deviation from unidimensionality as implied by CTT-based parameters such as I-UniCo, I-ECV, and IREAL. Moreover, these two items also had poor communality. Therefore, future studies should explore modification of these items for improvement in these psychometric measures.

The three threshold levels for all the 21 items were ordered as required. Moreover, the gap between two consecutive response levels were consistent and constant, i.e., 0.78 for τi1- τi2, and 0.66–0.67 for τi2- τi3 [48]. This consistency in expected response pattern levels of item scores is also visibly clear in the ICCs. Therefore, the response pattern for the individual items of the DASS-21 is appropriate [49]. Similarly, a study among Australian adults showed that the DASS-21 items had ordered threshold levels [49]. The width of spread of the latent construct (i.e., general distress) did not match the spread of the item difficulty level [50]. This disparity in the DASS-21 Wright map may be related to the non-clinical nature, and small sample size of the study population.

Finally, in the present study, item-level invariance was noted for all the DASS-21 items across gender groups. Similarly, item-level invariance for the DASS-21 was observed among Iranian medical students for all but one item [51]. In summary, the robust IRT measures also favored validity of the DASS-21 in the study population.

### Limitations

It is important to emphasize specific limitations when interpreting the present study’s findings. While examining generalizability, it is important to bear in mind the scope of limitations borne out by the specific sampling strategy and study setting. A larger and more gender-balanced sample might have provided a more comprehensive understanding of the psychometric properties of the DASS-21 in this context. Nevertheless, it is important to acknowledge that the study’s sample size was sufficient. Moreover, the lack of substantial skewness and kurtosis issues further supported the representativeness of the sample used in the present study. Moreover, the present study employed rigorous psychometric validity testing methods, incorporating both classical theory and Rasch rating theory parameters. The lack of clinical diagnosis to assess the concurrent validity, non-implementation of multi-group CFA, temporal CFA, and test-re-test reliability are other limitations. Future studies should explore these characteristics. This study employed the original English version of the DASS-21. Even though the study sample comprised of university students with the expected level of proficiency in English, some symptom concepts as mentioned in the Western-developed DASS-21 may not fully align with local expressions of distress.

### Conclusion

The psychometric validity of the DASS-21 was found to be robust, as evidenced by the analysis conducted using classical and item response theory measures among Ethiopian university students. There is much psychometric novelty in the present study. The study (i) is the first to assess measures of quality and effectiveness of factor score estimates, and construct applicability of the DASS-21, (ii) is one of the few with detailed assumption testing of the factor analysis on DASS-21 (i.e., testing univariate/multivariate normality assumptions, and using a method relevant to the specific data type, on the polychoric correlation matrix after checking KMO, Barttlett’s test, determinants, and communality), (iii) comprises one of the most detailed item-level psychometric testing using both CTT and IRT measures, (iv) is the first examining DASS-21 psychometrics to discuss in detail why two to three of the items were not deleted even though these had sub-optimal values for some of the parameters, (v) used a very extensive list of IRT-based analysis plans, and (vi) is the first to evaluate the original English version of the DASS-21 among Ethiopians. This is important because more than 80 languages and language-related ethnicities exist in Ethiopia. Many Ethiopian students cannot read or write proficiently in the official language (Amharic).

## Supporting information

S1 File Supplementary figures and tables. This file includes Figure S1–S2 showing parallel analysis and Item characteristic curves of DASS-21, and Table S1–S3(a-d) detailing inter-item polychoric correlation matrix, sample size adequacy, quality and effectiveness of factor score, construct replicability and reliability, and factor loading values of different models of DASS-21.
(DOCX)

S2 File Inclusivity in global research. This file includes a table summarizing country representation and author contribution diversity, in line with PLOS’ inclusivity guidelines.
(DOCX)

## References

[1] Asif S, Mudassar A, Shahzad TZ, Raouf M, Pervaiz T. Frequency of depression, anxiety and stress among university students. Pak J Med Sci. 2020;36(5):971–6. doi: 10.12669/pjms.36.5.1873 32704273 PMC7372668

[2] Griggs S. Hope and mental health in young adult college students: an integrative review. J Psychosoc Nurs Ment Health Serv. 2017;55(2):28–35. doi: 10.3928/02793695-20170210-04 28218927

[3] Othman N, Ahmad F, El Morr C, Ritvo P. Perceived impact of contextual determinants on depression, anxiety and stress: a survey with university students. Int J Ment Health Syst. 2019;13:17. doi: 10.1186/s13033-019-0275-x 30962817 PMC6434867

[4] Lipson SK, Zhou S, Abelson S, Heinze J, Jirsa M, Morigney J, et al. Trends in college student mental health and help-seeking by race/ethnicity: Findings from the national healthy minds study, 2013-2021. J Affect Disord. 2022;306:138–47. doi: 10.1016/j.jad.2022.03.038 35307411 PMC8995361

[5] Eisenberg D, Golberstein E, Hunt JB. Mental Health and academic success in college. The BE Journal of Economic Analysis & Policy. 2009;9(1). doi: 10.2202/1935-1682.2191

[6] Abdulghani HM, AlKanhal AA, Mahmoud ES, Ponnamperuma GG, Alfaris EA. Stress and its effects on medical students: a cross-sectional study at a college of medicine in Saudi Arabia. J Health Popul Nutr. 2011;29(5):516–22. doi: 10.3329/jhpn.v29i5.8906 22106758 PMC3225114

[7] Hysenbegasi A, Hass SL, Rowland CR. The impact of depression on the academic productivity of university students. J Ment Health Policy Econ. 2005;8(3):145–51. 16278502

[8] Mofatteh M. Risk factors associated with stress, anxiety, and depression among university undergraduate students. AIMS Public Health. 2020;8(1):36–65. doi: 10.3934/publichealth.2021004 33575406 PMC7870388

[9] BestColleges. College mental health statistics. Available from: https://www.bestcolleges.com/research/college-student-mental-health-statistics/#fn-1. 2023.

[10] Lovibond SH, Lovibond PF. Manual for the Depression Anxiety & Stress Scales. 2nd ed. Psychology Foundation. 1995.

[11] Lovibond PF, Lovibond SH. The structure of negative emotional states: comparison of the Depression Anxiety Stress Scales (DASS) with the beck depression and anxiety inventories. Behav Res Ther. 1995;33(3):335–43. doi: 10.1016/0005-7967(94)00075-u 7726811

[12] Makara-Studzińska M, Tyburski E, Załuski M, Adamczyk K, Mesterhazy J, Mesterhazy A. Confirmatory factor analysis of three versions of the Depression Anxiety Stress Scale (DASS-42, DASS-21, and DASS-12) in polish adults. Front Psychiatry. 2022;12:770532. doi: 10.3389/fpsyt.2021.770532 35058818 PMC8764392

[13] Lee D. The convergent, discriminant, and nomological validity of the Depression Anxiety Stress Scales-21 (DASS-21). J Affective Disorders. 2019;259:136–42. doi: 10.1016/j.jad.2019.06.036 31445339

[14] Wardenaar KJ, Wanders RBK, Jeronimus BF, de Jonge P. The psychometric properties of an internet-administered version of the Depression Anxiety and Stress Scales (DASS) in a Sample of Dutch Adults. J Psychopathol Behav Assess. 2017;40(2):318–33. doi: 10.1007/s10862-017-9626-6 29937624 PMC5978836

[15] Hamilton M. The assessment of anxiety states by rating. Br J Med Psychol. 1959;32(1):50–5. doi: 10.1111/j.2044-8341.1959.tb00467.x 13638508

[16] Manzar MD, BaHammam AS, Hameed UA, Spence DW, Pandi-Perumal SR, Moscovitch A, et al. Dimensionality of the pittsburgh sleep quality index: a systematic review. Health Qual Life Outcomes. 2018;16(1):89. doi: 10.1186/s12955-018-0915-x 29743066 PMC5944037

[17] Manzar MD, Jahrami HA, Bahammam AS. Structural validity of the Insomnia Severity Index: A systematic review and meta-analysis. Sleep Med Rev. 2021;60:101531. doi: 10.1016/j.smrv.2021.101531 34428679

[18] Kyriazos TA. Applied psychometrics: sample size and sample power considerations in factor analysis (EFA, CFA) and SEM in general. Psychology. 2018;9(08):2207.

[19] Lorenzo-Seva U, Ferrando PJ. Robust Promin: Un método para la rotación de factores de diagonal ponderada. Liberabit. 2019;25(1):99–106.

[20] Lorenzo-Seva U, Van Ginkel JR. Imputación múltiple de valores perdidos en el análisis factorial exploratorio de escalas multidimensionales: estimación de las puntuaciones de rasgos latentes. Anales de Psicología. 2016;32(2):596–608.

[21] Lorenzo-Seva U, Timmerman ME, Kiers HAL. The hull method for selecting the number of common factors. Multivariate Behav Res. 2011;46(2):340–64. doi: 10.1080/00273171.2011.564527 26741331

[22] Hu L, Bentler PM. Cutoff criteria for fit indexes in covariance structure analysis: Conventional criteria versus new alternatives. Structural Equation Modeling: A Multidisciplinary Journal. 1999;6(1):1–55. doi: 10.1080/10705519909540118

[23] Ferrando PJ, Lorenzo-Seva U. Assessing the quality and appropriateness of factor solutions and factor score estimates in exploratory item factor analysis. Educ Psychol Meas. 2018;78(5):762–80. doi: 10.1177/0013164417719308 32655169 PMC7328234

[24] Hancock GR, Mueller RO. (2000). Rethinking construct reliability within latent variable systems. In Cudek R, du Toit SHC, Sorbom DF (Eds.), Structural equation modeling: Present and future (pp. 195–216). Lincolnwood, IL: Scientific Software.

[25] Andrich D. A rating formulation for ordered response categories. Psychometrika. 1978;43(4):561–73.

[26] Hladká A, Martinková P. difNLR: Generalized logistic regression models for DIF and DDF detection. R Journal. 2020;12(1):300–23.

[27] Lim CR, Harris K, Dawson J, Beard DJ, Fitzpatrick R, Price AJ. Floor and ceiling effects in the OHS: an analysis of the NHS PROMs data set. BMJ Open. 2015;5(7):e007765. doi: 10.1136/bmjopen-2015-007765 26216152 PMC4521553

[28] Manzar MdD, Albougami A, Salahuddin M, Sony P, Spence DW, Pandi-Perumal SR. The Mizan meta-memory and meta-concentration scale for students (MMSS): a test of its psychometric validity in a sample of university students. BMC Psychol. 2018;6(1). doi: 10.1186/s40359-018-0275-7 PMC6299649 30563573

[29] Holgado–Tello FP, Chacón–Moscoso S, Barbero–García I, Vila–Abad E. Polychoric versus Pearson correlations in exploratory and confirmatory factor analysis of ordinal variables. Qual Quant. 2008;44(1):153–66. doi: 10.1007/s11135-008-9190-y

[30] Muthen B, Kaplan D. A comparison of some methodologies for the factor analysis of non‐normal Likert variables: A note on the size of the model. Brit J Math & Statis. 1992;45(1):19–30. doi: 10.1111/j.2044-8317.1992.tb00975.x

[31] Kakemam E, Navvabi E, Albelbeisi AH, Saeedikia F, Rouhi A, Majidi S. Psychometric properties of the Persian version of Depression Anxiety Stress Scale-21 Items (DASS-21) in a sample of health professionals: a cross-sectional study. BMC Health Serv Res. 2022;22(1):111. doi: 10.1186/s12913-022-07514-4 35078477 PMC8789546

[32] Bond TG, Fox CM. Applying the Rasch model: Fundamental measurement in the human sciences. 3rd ed. Psychology Press. 2013.

[33] Manzar MD, Salahuddin M, Khan TA, Shah SA, Mohammad NS, Nureye D, et al. Psychometric properties of a brief metamemory and metaconcentration scale in substance use problem. Int J Mental Health and Addiction. 2021;19(5):1690–704. doi: 10.1007/s11469-020-00319-6

[34] Lorenzo-Seva U, Ferrando PJ. FACTOR: a computer program to fit the exploratory factor analysis model. Behav Res Methods. 2006;38(1):88–91. doi: 10.3758/bf03192753 16817517

[35] Ali AM, Green J. Factor structure of the depression anxiety stress Scale-21 (DASS-21): Unidimensionality of the Arabic version among Egyptian drug users. Subst Abuse Treat Prev Policy. 2019;14(1):40. doi: 10.1186/s13011-019-0226-1 31533766 PMC6751677

[36] Martins BG, Silva WRD, Maroco J, Campos JADB. Escala de depressão, ansiedade e estresse: propriedades psicométricas e prevalência das afetividades. Jornal Brasileiro de Psiquiatria. 2019;68:32–41.

[37] Comrey AL, & Lee HB. (1992). A first course in factor analysis (2nd ed.). Lawrence Erlbaum Associates.

[38] Camacho Á, Cordero ED, Perkins T. Psychometric Properties of the DASS-21 among latina/o college students by the US-Mexico border. J Immigr Minor Health. 2016;18(5):1017–23. doi: 10.1007/s10903-016-0415-1 27037556

[39] Patrick J, Dyck M, Bramston P. Depression Anxiety Stress Scale: is it valid for children and adolescents?. J Clin Psychol. 2010;66(9):996–1007. doi: 10.1002/jclp.20696 20694962

[40] Zanon C, Brenner RE, Baptista MN, Vogel DL, Rubin M, Al-Darmaki FR, et al. Examining the dimensionality, reliability, and invariance of the Depression, Anxiety, and Stress Scale-21 (DASS-21) across eight countries. Assessment. 2021;28(6):1531–44. doi: 10.1177/1073191119887449 31916468

[41] De Champlain AF. A primer on classical test theory and item response theory for assessments in medical education. Med Educ. 2010;44(1):109–17. doi: 10.1111/j.1365-2923.2009.03425.x 20078762

[42] Albougami A, Manzar MD. Insomnia severity index: a psychometric investigation among Saudi nurses. Sleep Breath. 2019;23(3):987–96. doi: 10.1007/s11325-019-01812-8 30850944

[43] George D, Mallery P. SPSS for Windows step by step: A simple guide and reference. 4th ed. Allyn & Bacon. 2003.

[44] Kim H-Y. Statistical notes for clinical researchers: assessing normal distribution (2) using skewness and kurtosis. Restor Dent Endod. 2013;38(1):52–4. doi: 10.5395/rde.2013.38.1.52 23495371 PMC3591587

[45] Trizano-Hermosilla I, Alvarado JM. Best alternatives to cronbach’s alpha reliability in realistic conditions: congeneric and asymmetrical measurements. Front Psychol. 2016;7:769. doi: 10.3389/fpsyg.2016.00769 27303333 PMC4880791

[46] Yang FM, Kao ST. Item response theory for measurement validity. Shanghai Arch Psychiatry. 2014;26(3):171–7. doi: 10.3969/j.issn.1002-0829.2014.03.010 25114494 PMC4118016

[47] Kook SH, Varni JW. Validation of the Korean version of the pediatric quality of life inventory 4.0 (PedsQL) generic core scales in school children and adolescents using the Rasch model. Health Qual Life Outcomes. 2008;6:41. doi: 10.1186/1477-7525-6-41 18518951 PMC2459148

[48] Wind S, Hua C. Rasch measurement theory analysis in R: Illustrations and practical guidance for researchers and practitioners. Bookdown.org. 2021.

[49] Shea TL, Tennant A, Pallant JF. Rasch model analysis of the Depression, Anxiety and Stress Scales (DASS). BMC Psychiatry. 2009;9:21. doi: 10.1186/1471-244X-9-21 19426512 PMC2689214

[50] Planinic M, Boone WJ, Susac A, Ivanjek L. Rasch analysis in physics education research: Why measurement matters. Phys Rev Phys Educ Res. 2019;15(2). doi: 10.1103/physrevphyseducres.15.020111

[51] Jafari P, Nozari F, Ahrari F, Bagheri Z. Measurement invariance of the Depression Anxiety Stress Scales-21 across medical student genders. Int J Med Educ. 2017;8:116–22. doi: 10.5116/ijme.58ba.7d8b 28362630 PMC5376494

