# Supplementary material for: Depression, Anxiety, and Stress Scale-21 (DASS-21): Further psychometric exploration using robust item response theory and classical theory measures among university students
Source: PLoS One. 2025 Jul 28;20(7):e0325238. doi: 10.1371/journal.pone.0325238 (PMC12303295; doi:10.1371/journal.pone.0325238)
Supplement: S1 File — This file includes Figure S1–S2 showing parallel analysis and Item characteristic curves of DASS-21, and Table S1–S3(a-d) detailing inter-item polychoric correlation matrix, sample size adequacy, quality and effectiveness of factor score, construct replicability and reliability, and factor loading values of different models of DASS-21. (DOCX) [file pone.0325238.s001.docx]

Table S1 Inter-item polychoric correlation matrix of the Depression Anxiety Stress (DASS-21) scores in university students

|  | D-1 | D-2 | D-3 | D-4 | D-5 | D-6 | D-7 | D-8 | D-9 | D-10 | D-11 | D-12 | D-13 | D-14 | D-15 | D-16 | D-17 | D-18 | D-19 | D-20 | D-21 |
| --- | --- | --- | --- | --- | --- | --- | --- | --- | --- | --- | --- | --- | --- | --- | --- | --- | --- | --- | --- | --- | --- |
| D-1 | 1.00 |  |  |  |  |  |  |  |  |  |  |  |  |  |  |  |  |  |  |  |  |
| D-2 | 0.46 | 1.00 |  |  |  |  |  |  |  |  |  |  |  |  |  |  |  |  |  |  |  |
| D-3 | 0.40 | 0.29 | 1.00 |  |  |  |  |  |  |  |  |  |  |  |  |  |  |  |  |  |  |
| D-4 | 0.36 | 0.35 | 0.28 | 1.00 |  |  |  |  |  |  |  |  |  |  |  |  |  |  |  |  |  |
| D-5 | 0.38 | 0.31 | 0.31 | 0.48 | 1.00 |  |  |  |  |  |  |  |  |  |  |  |  |  |  |  |  |
| D-6 | 0.34 | 0.33 | 0.27 | 0.38 | 0.36 | 1.00 |  |  |  |  |  |  |  |  |  |  |  |  |  |  |  |
| D-7 | 0.38 | 0.23 | 0.20 | 0.48 | 0.40 | 0.30 | 1.00 |  |  |  |  |  |  |  |  |  |  |  |  |  |  |
| D-8 | 0.43 | 0.33 | 0.32 | 0.41 | 0.38 | 0.40 | 0.41 | 1.00 |  |  |  |  |  |  |  |  |  |  |  |  |  |
| D-9 | 0.40 | 0.23 | 0.24 | 0.43 | 0.46 | 0.36 | 0.49 | 0.43 | 1.00 |  |  |  |  |  |  |  |  |  |  |  |  |
| D-10 | 0.46 | 0.26 | 0.33 | 0.48 | 0.41 | 0.29 | 0.38 | 0.39 | 0.39 | 1.00 |  |  |  |  |  |  |  |  |  |  |  |
| D-11 | 0.41 | 0.27 | 0.37 | 0.41 | 0.54 | 0.34 | 0.40 | 0.40 | 0.51 | 0.48 | 1.00 |  |  |  |  |  |  |  |  |  |  |
| D-12 | 0.31 | 0.16 | 0.19 | 0.39 | 0.39 | 0.34 | 0.33 | 0.35 | 0.38 | 0.44 | 0.50 | 1.00 |  |  |  |  |  |  |  |  |  |
| D-13 | 0.43 | 0.31 | 0.23 | 0.43 | 0.35 | 0.43 | 0.44 | 0.41 | 0.44 | 0.43 | 0.47 | 0.45 | 1.00 |  |  |  |  |  |  |  |  |
| D-14 | 0.48 | 0.23 | 0.16 | 0.38 | 0.41 | 0.32 | 0.40 | 0.47 | 0.58 | 0.37 | 0.38 | 0.49 | 0.46 | 1.00 |  |  |  |  |  |  |  |
| D-15 | 0.46 | 0.25 | 0.32 | 0.50 | 0.40 | 0.32 | 0.51 | 0.37 | 0.46 | 0.54 | 0.52 | 0.46 | 0.50 | 0.43 | 1.00 |  |  |  |  |  |  |
| D-16 | 0.40 | 0.23 | 0.26 | 0.37 | 0.36 | 0.40 | 0.42 | 0.36 | 0.33 | 0.50 | 0.36 | 0.38 | 0.40 | 0.38 | 0.51 | 1.00 |  |  |  |  |  |
| D-17 | 0.34 | 0.18 | 0.28 | 0.43 | 0.36 | 0.30 | 0.38 | 0.33 | 0.41 | 0.46 | 0.47 | 0.31 | 0.44 | 0.44 | 0.45 | 0.41 | 1.00 |  |  |  |  |
| D-18 | 0.37 | 0.25 | 0.23 | 0.40 | 0.33 | 0.35 | 0.44 | 0.40 | 0.44 | 0.38 | 0.40 | 0.41 | 0.53 | 0.52 | 0.42 | 0.43 | 0.54 | 1.00 |  |  |  |
| D-19 | 0.35 | 0.26 | 0.22 | 0.31 | 0.37 | 0.26 | 0.47 | 0.32 | 0.36 | 0.36 | 0.32 | 0.32 | 0.43 | 0.40 | 0.40 | 0.36 | 0.37 | 0.43 | 1.00 |  |  |
| D-20 | 0.41 | 0.19 | 0.29 | 0.41 | 0.47 | 0.39 | 0.40 | 0.41 | 0.47 | 0.41 | 0.44 | 0.42 | 0.50 | 0.49 | 0.46 | 0.47 | 0.42 | 0.50 | 0.49 | 1.00 |  |
| D-21 | 0.41 | 0.18 | 0.23 | 0.39 | 0.45 | 0.42 | 0.42 | 0.32 | 0.50 | 0.46 | 0.52 | 0.44 | 0.49 | 0.44 | 0.48 | 0.50 | 0.44 | 0.41 | 0.38 | 0.61 | 1.00 |

D-1 to D-21: items of the DASS-21

Table S2 Multivariate descriptive, sample size adequacy, quality and effectiveness of factor score, construct replicability and reliability measures of the Depression Anxiety Stress (DASS-21) scale scores in university students

| Measures | Values |
| --- | --- |
| Multivariate descriptive  Mardia’s skewness  Mardia’s kurtosis | Χ^2^(df=1771)= 3773.485, p=1.00  Χ^2^= 29.923, p<.001 |
| Quality and effectiveness of factor score estimates^*^  Factor Determinacy Index (FDI)  expected a posteriori marginal reliability  Sensitivity ratio (SR)  Expected percentage of true differences (EPTD) | 0.967  0.936  3.825  94.4% |
| Construct replicability^*^  H-Latent  H-Observed | 0.936  0.917 |
| Sample size adequacy  Bartlett’s test of Sphericity  Determinant  KMO (95% confidence interval) | Χ^2^(df=210)= 3160.9, p<0.001  0.00007  0.938 (0.887-0.953) |
| Internal consistency  Cronbach’s alpha  McDonald's Omega | 0.93  0.93 |

Kaiser-Meyer-Olkin Test of Sampling Adequacy (KMO)

^*^ For the 1-Factor structure of the DASS-21

Table S3a. Factor loading values for the 1-Factor model the Depression, Anxiety and Stress Scale - 21 Items (DASS-21) in the Ethiopian university students


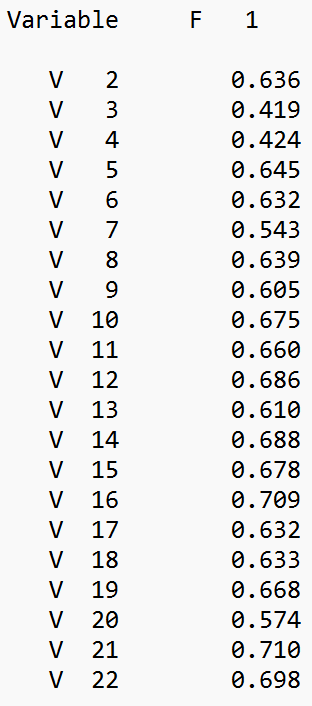


Table S3b. Factor loading values for the 2-Factor model the Depression, Anxiety and Stress Scale - 21 Items (DASS-21) in the Ethiopian university students


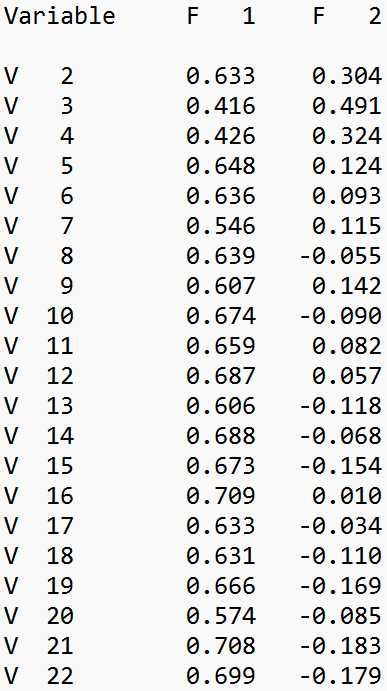


Table S3c. Factor loading values for the 3-Factor model the Depression, Anxiety and Stress Scale - 21 Items (DASS-21) in the Ethiopian university students


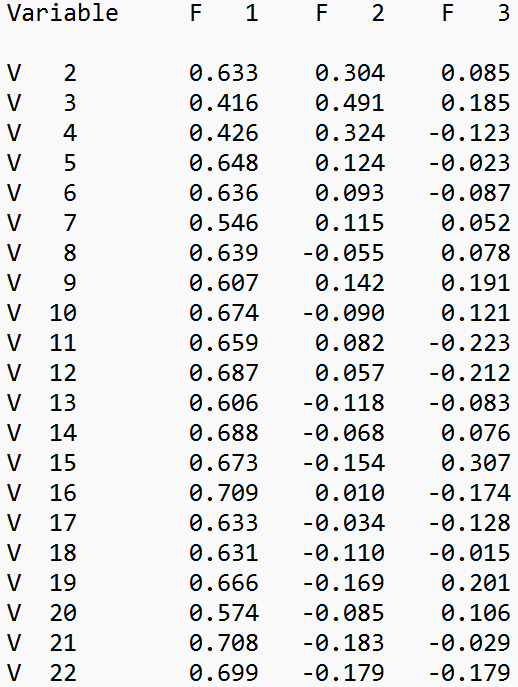


Table S3d. Factor loading values for the bifactor of the Depression, Anxiety and Stress Scale - 21 Items (DASS-21) in the Ethiopian university students

| 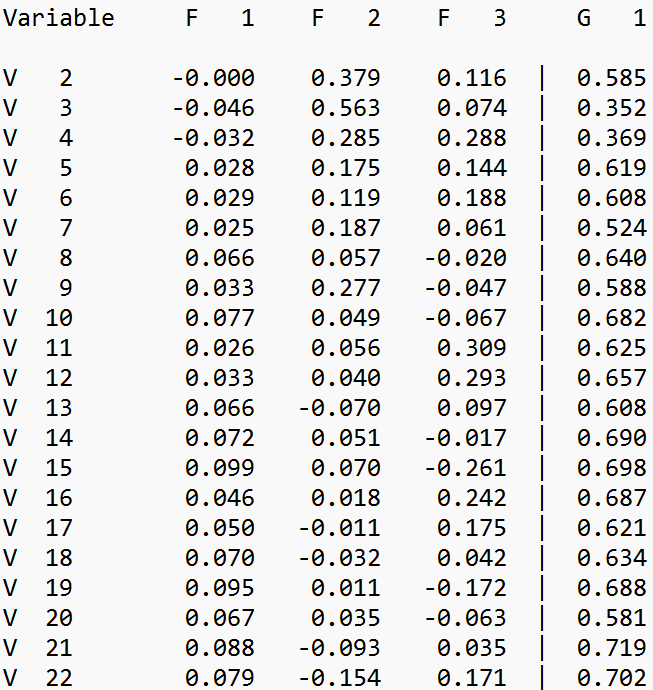 | V2 to V22: Items of the DASS-21 scale; F 1: Factor-1, F 2: Factor-2, F 3: Factor-3, G-1: Second order first factor  All loading values are for unrotated solutions. |
| --- | --- |


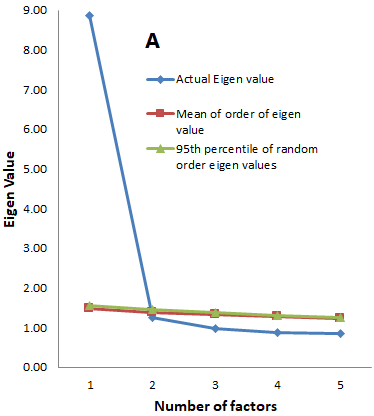

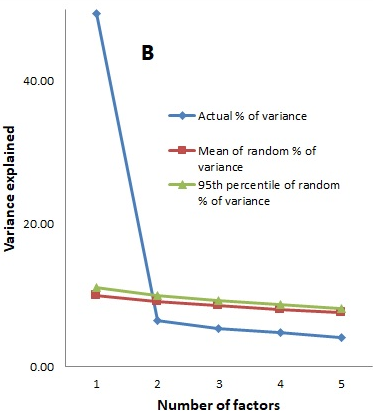


Figure S1 Parallel analysis based on: (A) principal component analysis, and (B) minimum rank factor analysis of the Depression Anxiety Stress-21 (DASS-21) scale scores in university students.

##
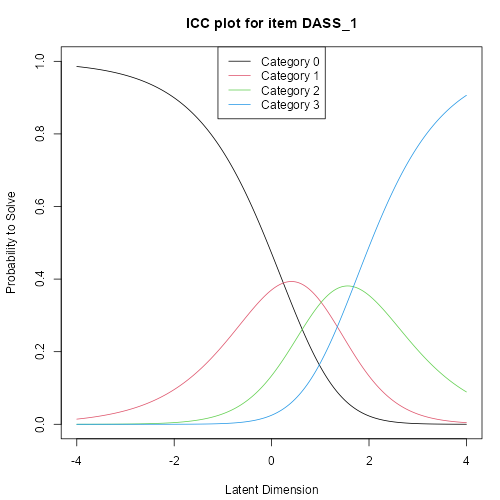

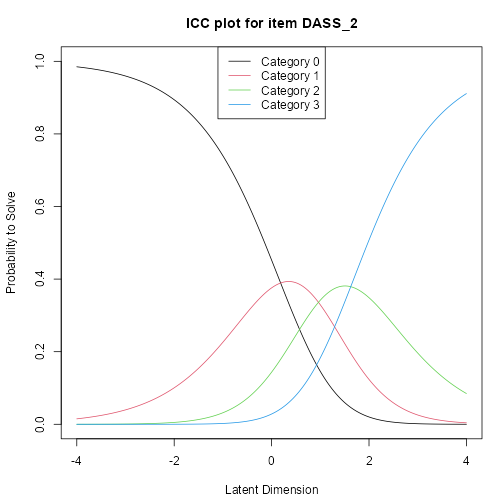

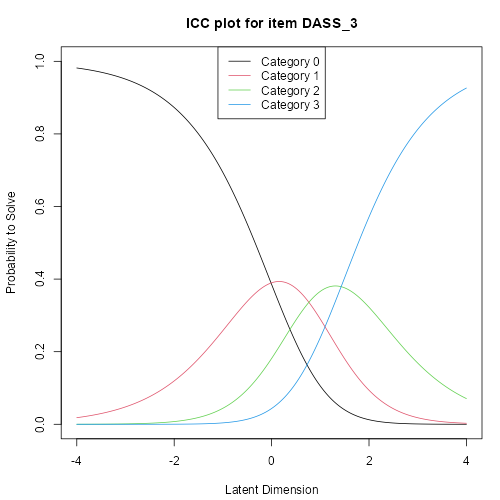

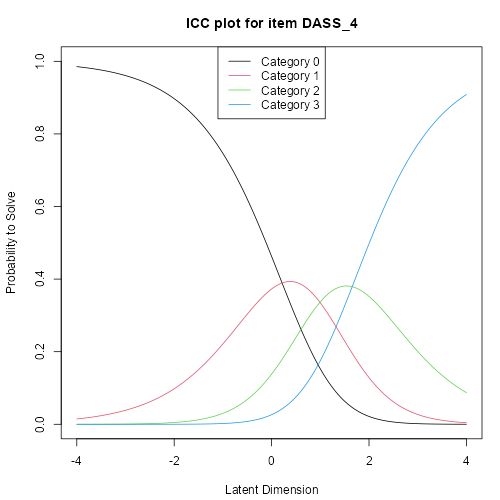

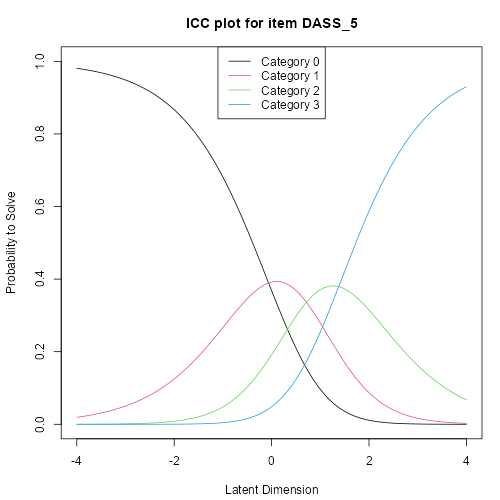

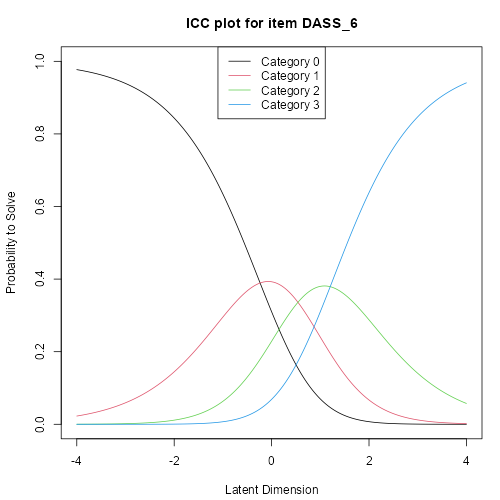

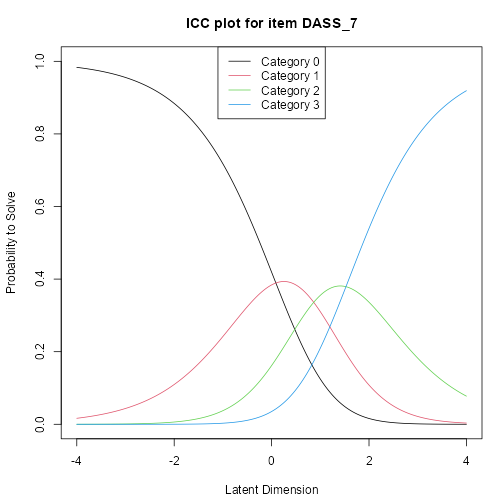

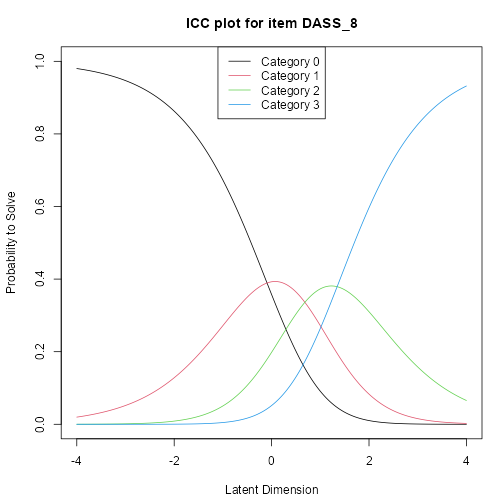

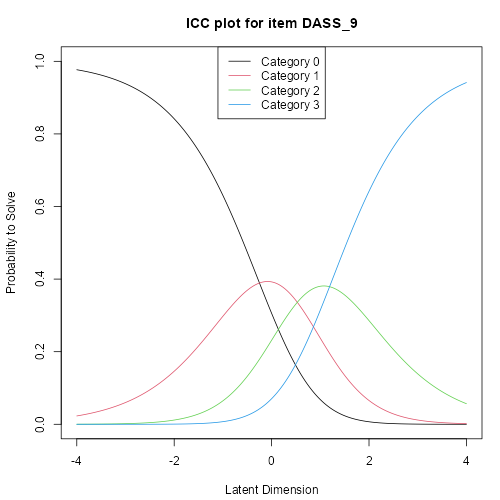

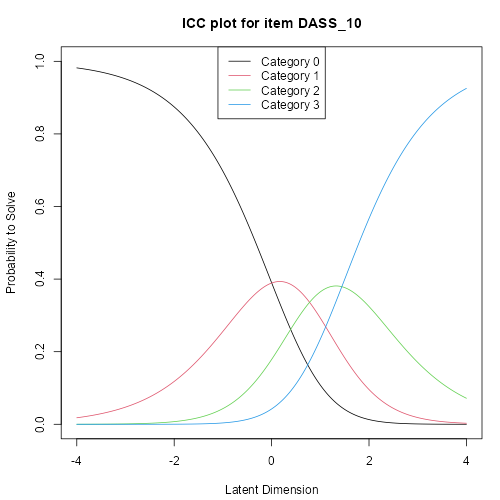

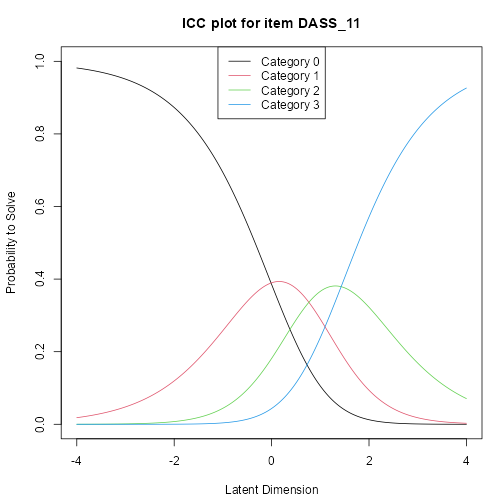

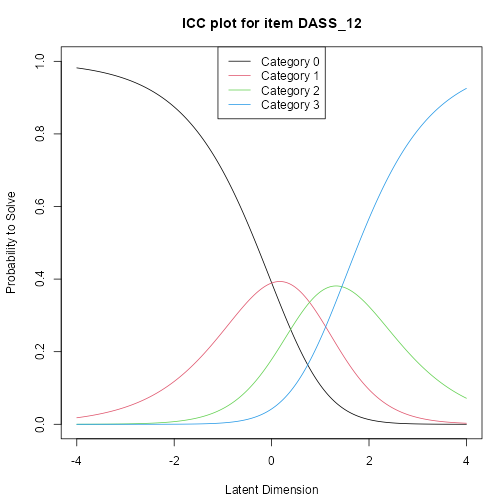


##
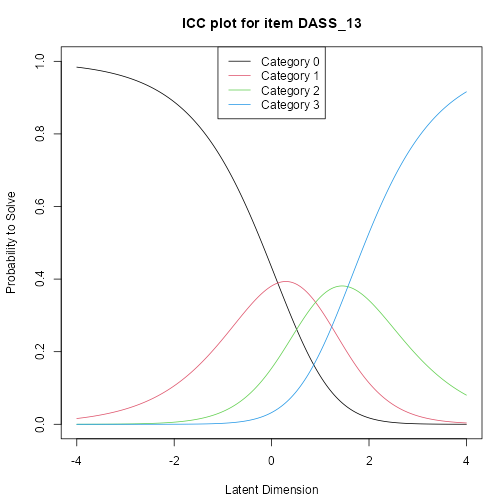

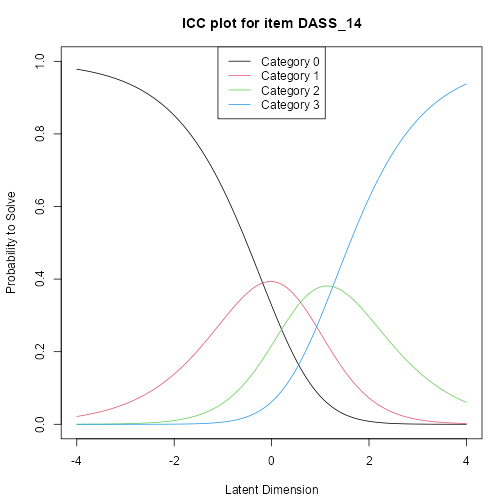

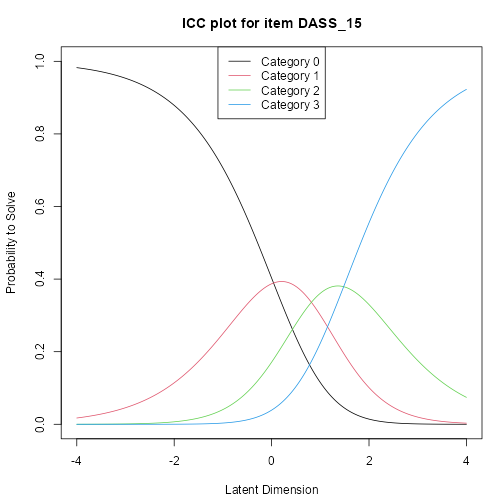

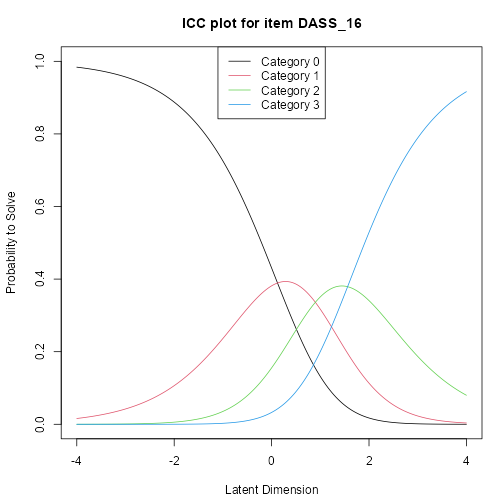

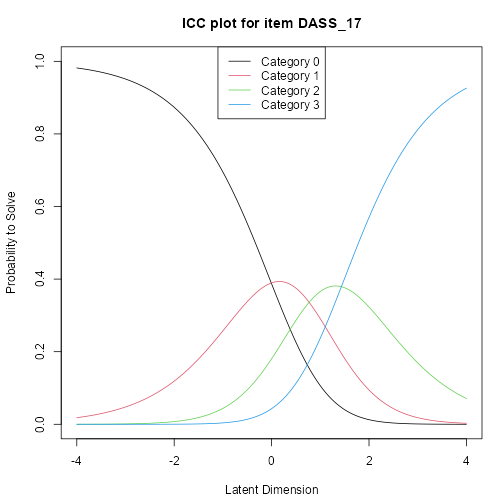

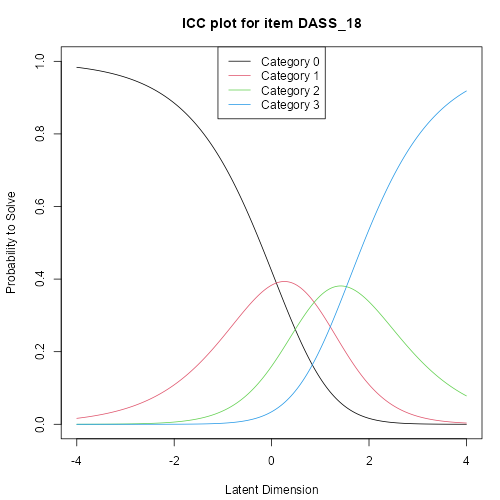


##
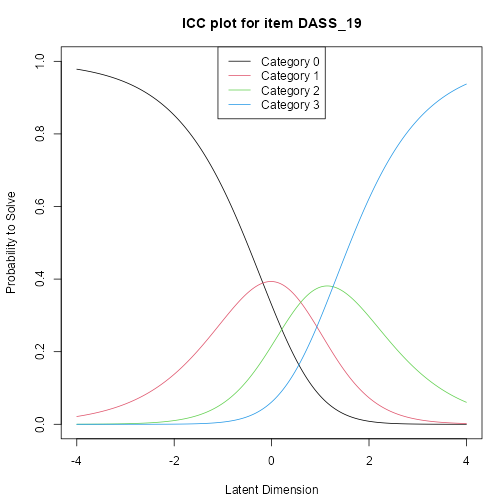

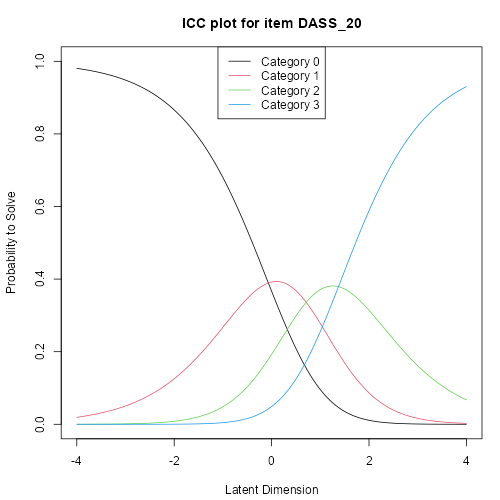

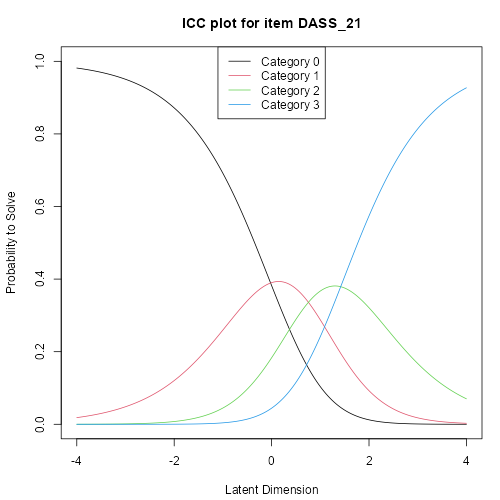


Figure S2. Item characteristic curves of the Depression Anxiety Stress (DASS-21) scale scores among university students
